# Investigating biological traces of traumatic stress in changing societies: challenges and directions from the ESTSS Task Force on Neurobiology

**DOI:** 10.3402/ejpt.v7.29453

**Published:** 2016-03-18

**Authors:** Kathleen Thomaes, Carien de Kloet, Sarah Wilker, Wissam El-Hage, Ingo Schäfer, Birgit Kleim, Christian Schmahl, Mirjam van Zuiden

**Affiliations:** 1 GGZ inGeest/Department of Psychiatry & Anatomy and neurosciences, VU University Medical Center, Amsterdam, The Netherlands; 2 Arq Psychotrauma Expert Group, Foundation Centrum '45, Oegstgeest, The Netherlands; 3 Department of Clinical and Biological Psychology, Ulm University, Ulm, Germany; 4 Department of Psychiatry, CHRU de Tours, Inserm U930, University of Tours, Tours, France; 5 Department of Psychiatry, University Medical Center Hamburg-Eppendorf, University of Hamburg, Hamburg, Germany; 6 Department of Psychology, University of Zurich, Zürich, Switzerland; 7 Department of Psychiatry, Psychotherapy and Psychosomatics, Psychiatric University Hospital Zurich, Zurich, Switzerland; 8 Department of Psychosomatic Medicine and Psychotherapy, Central Institute of Mental Health, Medical Faculty Mannheim, Heidelberg University, Heidelberg, Germany; 9 Department of Psychiatry, Academic Medical Center, University of Amsterdam, Amsterdam, The Netherlands

**Keywords:** Trauma, PTSD, vulnerability, resilience, neurobiology

## Abstract

Traumatic stress can have severe consequences for both mental and physical health. Furthermore, both psychological and biological traces of trauma increase as a function of accumulating traumatic experiences. Neurobiological research may aid in limiting the impact of traumatic stress, by leading to advances in preventive and treatment interventions. To promote the possibility for clinical implementation of novel research findings, this brief review describes timely conceptual and methodological challenges and directions in neurobiological trauma research on behalf of the Task Force “Neurobiology of Traumatic Stress” of the European Society for Traumatic Stress Studies (ESTSS). The most important conceptual challenges are the heterogeneity of disorders and existence of subtypes across diagnostic categories: differential latent profiles and trajectories regarding symptom expression and neural correlates are being unraveled; however, similar latent classes’ approaches for treatment response and neurobiological data remain scarce thus far. The key to improving the efficacy of currently available preventive interventions and treatments for trauma-related disorders lies in a better understanding and characterization of individual differences in response to trauma and interventions. This could lead to personalized treatment strategies for trauma-related disorders, based on objective information indicating whether individuals are expected to benefit from them. The most important methodological challenge identified here is the need for large consortia and meta-analyses or, rather, mega-analyses on existent data as a first step. In addition, large multicenter studies, combining novel methods for repeated sampling with more advanced statistical modeling techniques, such as machine learning, should aim to translate identified disease mechanisms into molecular blood-based biomarker combinations to predict disorder vulnerability and treatment responses.

In the early 2000s, over 60% of Western European adults reported at least one potentially traumatic experience, ranging from accidents and disasters to interpersonal violence and war exposure (Darves-Bornoz et al., 2008). Since then, war crimes and terror against civilians have been persistent problems. Current European examples are the East-Ukraine Crisis, including the shooting of passenger flight MH17, and the terrorist attacks on November 13, 2015, in Paris, France. Even more, ongoing conflicts outside Europe lead to a high number of trauma survivors seeking asylum in Europe. As of November 2015, an unprecedented total of over 500,000 refugees reached Europe by Sea. Accordingly, traumatic experiences in and outside of Europe will likely have long lasting influences on European society. A central challenge will be the (secondary) prevention and treatment of the adverse consequences of these traumatic experiences.

While most traumatized individuals are resilient (Bonanno, Westphal, & Mancini, 2011), a significant subset develops trauma-related disorders, including posttraumatic stress disorder (PTSD) (Darves-Bornoz et al., 2008) and dissociative disorders (Teicher & Samson, 2013), and other psychiatric disorders such as depressive (North et al., 2015) and substance abuse disorders (Brady & Back, 2012). In the context of refugees, who often survived repeated trauma exposure, it is important to note that the risk for PTSD increases with the number of traumatic experiences (Kolassa, Kolassa, Ertl, Papassotiropoulos, & De Quervain, 2010). Furthermore, the probability of spontaneous remission from PTSD decreases with increasing traumatic load (Kolassa et al., 2010). The aim of this review paper is to outline current challenges in the field of neurobiological trauma research, as well as promising research directions that may help reducing the negative impact of trauma and may promote clinical implementation of novel research findings. For this purpose, we provide a concise overview of known biological consequences of trauma and PTSD; lines of research that may reduce the negative impact of trauma by improved prevention or treatment; conceptual and methodological challenges associated with these lines of research; and how the “ESTSS Task Force on Neurobiology of Traumatic Stress” aims to address some of the raised issues.

## Adverse biological consequences

Trauma and subsequent trauma-related disorders not only affect mental health but also have adverse consequences on the biological level. Both trauma and trauma-related disorders are associated with increased risk for age-related physical diseases (De Hert et al., 2011) and increased mortality (Chesney, Goodwin, & Fazel, 2014). Furthermore, the building block effect of traumatic load (Schauer et al., 2003) is also reflected in a dose-dependent increased risk for age-related physical diseases (Felitti et al., 1998).

Although the exact molecular mechanisms underlying this elevated risk and their interaction with lifestyle factors warrant further research, a multitude of studies have shown that both trauma exposure and trauma-related disorders are associated with dysfunctioning of numerous biological systems (Schmidt, Kaltwasser, & Wotjak, 2013). In addition, initial evidence indicates a cumulative effect of trauma load and PTSD symptom severity, as reflected by findings indicating premature aging of, for example, the immune system (Sommershof et al., 2009; Morath et al., 2014), and telomere length (Ladwig et al., 2013) as a function of traumatic load and PTSD severity. However, recent research also has shown that dysfunctioning in several biological systems precedes the development of trauma-related disorders (Schmidt et al., 2013). These biological vulnerabilities likely (partially) result from epigenetic changes due to prior traumatic experiences of the individual (Heinzelmann & Gill, 2013) or previous generations (Yehuda et al., 2014).

## Neurobiological research to limit the impact of trauma

To limit the impact of traumatic stress, it is pivotal to increase our knowledge on the neurobiological mechanisms preceding development of PTSD and other trauma-related disorders. Further unraveling peri- and posttrauma neurobiological (and related cognitive) mechanisms underlying development of trauma-related disorders may improve prevention in two ways. First, identification of risk biomarkers may aid in early identification of individuals at risk. This way, early interventions may be targeted toward at-risk individuals, thereby not interfering with normative adaptive recovery in most trauma-exposed. Second, identification of these mechanisms may inform development or improvement of preventive interventions (Van Zuiden et al., 2013). Several promising preventive interventions for PTSD have recently been developed. Two of the most promising interventions to be administered early upon trauma are prolonged exposure and single or repeated administration of hydrocortisone (for review, see Sijbrandij et al., 2015). However, none of these interventions have been implemented yet because large-scale replication and feasibility studies still need to be performed. Therefore, it remains important to develop additional preventive strategies and investigate who may benefit most from such interventions.

Once trauma-related disorders have become manifest, a large subset of patients seeking treatment does not adequately respond to the existing treatments (Bisson, Roberts, Andrew, Cooper, & Lewis, 2013). Neurobiological research on processes associated with successful recovery is essential in the development or improvement of effective treatment strategies. The identification of mechanisms underlying successful recovery may augment the efficacy of currently existing evidence-based psychotherapeutic interventions, by providing the opportunity to add neurobiological-informed agents that target processes found to be pivotal for successful treatment and recovery. Such strategies include pharmacological enhancement of psychotherapy (for review, see Dunlop, Mansson, & Gerardi, 2012) and combining psychotherapy with brain stimulation and neuromodulation techniques (for review, see Marin, Camprodon, Dougherty, & Milad, 2014). Currently, this field is still in its infancy, and only few neurobiological augmentation strategies have been investigated yet. Regarding medication-enhanced psychotherapy, addition of d-cycloserine, hydrocortisone, MDMA, and propranolol in addition to exposure therapy have been the most studied interventions. Seeing the promising effects in anxiety disorders, expectations regarding their efficacy in enhancing treatment–response were quite high. However, the current evidence for the efficacy of these agents is inconclusive, and larger RCT's are necessary to investigate whether these agents indeed hold promise (for reviews, see De Kleine, Rothbaum, Van Minnen, 2013, Ori et al., 2015). Also, the safety (for e.g., MDMA) and potential interactions with commonly used selective serotonin re-uptake inhibitors (SSRIs) and alcohol (for e.g., propranolol) should be investigated (De Kleine et al., 2013). Elucidation of neurobiological mechanisms of PTSD recovery has also spurred research on other potentially promising agents for medication-enhanced psychotherapy. For example, neural effects of intranasal oxytocin administration in PTSD patients indicate that this may also be a promising augmentation strategy, but this needs to be investigated within a clinical setting (Koch et al., 2015). Importantly, specific treatment interventions may only work for certain subgroups of patients because trauma-related disorders show large heterogeneity and individual treatment responses vary accordingly. Therefore, treatment response may be improved by identifying biomarkers to predict treatment response, for this could eventually lead to algorithms for personalized treatment. Several biological parameters have already been found to predict PTSD treatment response, for example: cytosine methylation; GR gene expression; 5-HTTLPR genotype; BDNF in serum; anterior cingulate cortex (ACC) volume and ACC and amygdala activity (for review, see Thomaes et al., 2014; Yehuda et al., 2013). Other parameters were found to change in parallel with recovery, for example: methylation of the FKBP5 gene, cortisol and DHEA levels, and ACC, insula and amygdala activity (Ibid). However, studies investigating such biomarkers were generally small, and none of these potential biomarkers have yet reached the threshold of a specific and clinically usable biomarker (Lehrner & Yehuda, 2014).

Over the past decades, much knowledge on neurobiological correlates of trauma and trauma-related disorders has been collected in cross-sectional studies comparing affected individuals to control groups, of which the most consistent conclusions are smaller hippocampal volume, increased amygdala activity to threat, sympathic nervous system hyperactivity and glucocorticoid receptor dysregulation in PTSD patients (for review, see Schmidt et al., 2013), although findings are majorly impacted by whether controls were trauma-exposed or not. More recently, prospective and longitudinal research in individuals at risk for trauma exposure, recently trauma-exposed individuals, and patients commencing treatment has also been initiated, as well as translational approaches integrating experimental rodent and human *in vivo* and *in vitro* studies and clinical trials (for review, see Schmidt et al., 2013). While these are promising novel directions, until now translation of findings into clinical practice remains limited. To achieve this, several timely challenges have to be overcome, of which we discuss the most important ones below.

## Conceptual challenges and directions

Driven by the classification of psychiatric disorders, much previous research on trauma-related disorders focused on one disorder at a time, while considering all patients as a homogenous sample. However, from a within-disorder perspective, it has become increasingly apparent that disorders are heterogeneous, and that subtypes regarding symptom expression and neurobiological correlates exist. This is supported by recent studies that identified differential latent profiles and trajectories for subsets of patients (e.g., Nugent, Koenen, & Bradley, 2012). However, similar latent class approaches for treatment response and neurobiological data remain scarce thus far (an exception is e.g., Galatzer-Levy et al., 2013). Also, moderating effects of essential patient characteristics, such as sex (including menstrual phase during neurobiological assessments, trauma exposure and/or treatment sessions), age, ethnicity, and developmental timing of trauma exposure on neurobiological mechanisms underlying development, recovery and treatment-mechanisms have only sparsely been addressed.

From a between-disorder perspective, the overlap in symptoms and vast comorbidity between disorders, as well as observed similar neurobiological correlates (e.g., short allele of serotonin transporter length polymorphism (Kuzelova, Ptacek, & Macek, 2010)), demonstrate that psychiatric disorders should not be regarded in isolation. Furthermore, the range of psychiatric disorders for which the onset and course is impacted by trauma exposure, especially when experienced early in life, is much broader than those formally acknowledged as trauma-related, including, for example, personality (Zanarini et al., 1997), depressive (North et al., 2015) psychotic (for review, see Varese et al., 2012), and bipolar disorders (for review, see Etain, Henry, Belivier, Mathieu, & Leboyer, 2008), stressing that research on neurobiological aspects of trauma should broaden its scope.

The key to improving the efficacy of currently available preventive interventions and treatments for trauma-related disorders lies in a better understanding and characterization of individual differences in response to trauma and interventions. This could lead to personalized treatment strategies for trauma-related disorders, based on objective information indicating whether individuals are expected to benefit from them. From both within- and between-disorder viewpoints, it can be argued that future studies should incorporate cross-disorder, trans-diagnostic, domain-oriented approaches at both the symptom and biological levels, as is also posited in the US National Institutes of Mental Health Research domain criteria (RDOC) framework (Insel et al., 2010). Also, whether findings in one patient population can be generalized toward other populations should receive more emphasis in future research, to ensure adequate translation of findings into effective clinical practice.

## Methodological challenges and directions

A large proportion of previous neurobiological studies had relatively small sample sizes, raising concerns about generalizability and validity of their results. Therefore, we need large consortia and meta-analyses or, rather, mega-analyses on existent data, such as the ENIGMA (Thompson et al., 2014) and Psychiatric Genomics Consortium (Psychiatric GWAS Consortium Steering Committee, 2009), as a first step. In addition, large multicenter studies, combining novel methods for repeated sampling, such as ecological momentary assessment via smartphones, with in-depth multilevel biological assessment may further advance the field. Current studies are often limited by focus on single biomarkers or other predictors as a basis for intervention allocation. However, we need more advanced statistical modeling techniques, such as machine learning (Sato et al., 2015), and should aim to translate identified disease mechanisms into molecular blood-based biomarker combinations (e.g., Chan et al., 2014) to combine different biological variables to predict disorder vulnerability and treatment responses. At the same time, the need for innovative pilot studies in single centers to develop and test novel hypotheses remains.

Replicability is another important point to be addressed within the field. Therefore, we need more transparent descriptions of analyses and data repositories, especially in neuroimaging studies where sample sizes are usually small and options of statistical analyses are large. Furthermore, with respect to replicability but also to data pooling, similar methodologies are needed to be able to compare results of individual studies, both regarding protocols used for collecting biological data and regarding questionnaires or interviews used for phenotypic assessment and assessment of trauma exposure.

In addition, non-clinical laboratory traumatic stress models may result in fundamental knowledge on the mechanisms underlying development of trauma-related disorders and on risk factors that may influence vulnerability upon trauma exposure. Such models, including the commonly used and well-validated trauma film paradigm that reliably induces mildly distressing intrusive re-experiencing (Holmes & Bourne, 2008), allow for detailed investigation of the exact temporal course of neurobiological responses during analogue traumatic stress, and how individual variability in neurobiological functioning relates to symptom development. This is important because such detailed assessment during trauma exposure is not feasible in clinical settings. Combining research in traumatized individuals with and without subsequent psychopathology with such experimental traumatic stress models (Ehring, Kleim, & Ehlers, 2011), but also with animal traumatic stress models and *in vitro* laboratory models may readily advance our fundamental knowledge on traumatic stress.

## Conclusions

As traumatic experiences increase the risk to suffer from psychiatric disorders, a central challenge for the European society is to timely prevent and treat trauma-related disorders. The exact neurobiological mechanisms underlying development and recovery of these disorders are as of yet not fully understood. To be able to limit the impact of traumatic stress on both an individual and societal level, it is pivotal to increase our knowledge of these neurobiological mechanisms.

In this review, we have described several conceptual and methodological challenges and directions for the research field. Within the ESTSS Task Force on “Neurobiology of Traumatic Stress,” we strive to address several of these issues in the following ways:

- Promote discussion and exchange of methodology between affiliated researchers
- Encourage publication of standardized assessment protocols and inform task force members about newly published protocols, via website and workshops or symposia on conferences on traumatic stress
- Provide a safe platform for researchers to investigate novel potential collaborations, including additional centers for multicenter trials, or novel avenues to test hypotheses in translational research
- As a first step in establishing novel collaborations, investigate possibilities to combine already collected neurobiological data from different research groups (e.g., mega-analysis and meta-analysis), specifically on the HPA axis

It is our hope and expectation that adequately addressing and overcoming the challenges highlighted in this review will promote the much needed development and implementation of novel neurobiological-informed preventive and personalized treatment strategies for trauma-related disorders.
